# (2-Hydroxy­benzoato-κ*O* ^1^)[tris­(1-methyl­benzimidazol-2-ylmethyl-κ*N* ^3^)amine-κ*N*]cobalt(II) perchlorate dimethyl­formamide sesquisolvate

**DOI:** 10.1107/S1600536808033758

**Published:** 2008-10-25

**Authors:** Xuyang Fan, Xingcai Huang, Kaitong Wang, Tao Sun, Huilu Wu

**Affiliations:** a School of Chemical and Biological Engineering, Lanzhou Jiaotong University, Lanzhou 730070, People’s Republic of China

## Abstract

In the title complex, [Co(C_7_H_5_O_3_)(C_27_H_27_N_7_)]ClO_4_·1.5C_3_H_7_NO, the Co^II^ ion is five-coordinated by four N atoms from a tris­(*N*-methyl­benzimidazol-2-ylmeth­yl)amine (Mentb) ligand and one O atom from a salicylate ligand in a distorted trigonal–bipyramidal geometry with approximate mol­ecular *C*_3_ symmetry. The perchlorate ion is disordered over two sites with equal occupancy. One dimethyl­formamide solvent mol­ecule lies on a general position and is disordered over two coplanar orientations with equal occupancy. A second dimethyl­formamide mol­ecule is disordered about a twofold rotation axis. There is an intra­molecular O—H⋯O hydrogen bond in the cation.

## Related literature

For related literature, see: Allen *et al.* (1987 ▶); Youngme *et al.* (2007 ▶).
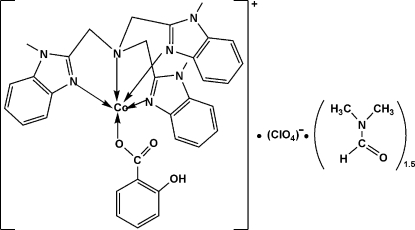


## Experimental

### 

#### Crystal data

[Co(C_7_H_5_O_3_)(C_27_H_27_N_7_)]ClO_4_·1.5C_3_H_7_NO
*M* *_r_* = 854.69
Monoclinic, 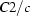

*a* = 27.7516 (5) Å
*b* = 11.4051 (2) Å
*c* = 25.0827 (5) Å
β = 102.9130 (10)°
*V* = 7738.1 (2) Å^3^
*Z* = 8
Mo *K*α radiation
μ = 0.58 mm^−1^
*T* = 153 (2) K
0.35 × 0.25 × 0.21 mm

#### Data collection

Rigaku R-Axis Spider diffractometer
Absorption correction: empirical (using intensity measurements) (*ABSCOR*; Higashi, 1995 ▶) *T*_min_ = 0.823, *T*_max_ = 0.888
36824 measured reflections
8867 independent reflections
6936 reflections with *I* > 2σ(*I*)
*R*_int_ = 0.029

#### Refinement

*R*[*F* ^2^ > 2σ(*F* ^2^)] = 0.039
*wR*(*F* ^2^) = 0.121
*S* = 1.00
8867 reflections
600 parameters
8 restraints
H atoms treated by a mixture of independent and constrained refinement
Δρ_max_ = 0.55 e Å^−3^
Δρ_min_ = −0.69 e Å^−3^

### 

Data collection: *RAPID-AUTO* (Rigaku/MSC, 2004 ▶); cell refinement: *RAPID-AUTO*; data reduction: *RAPID-AUTO*; program(s) used to solve structure: *SHELXS97* (Sheldrick, 2008 ▶); program(s) used to refine structure: *SHELXL97* (Sheldrick, 2008 ▶); molecular graphics: *SHELXTL* (Sheldrick, 2008 ▶) and *PLATON* (Spek, 2003 ▶); software used to prepare material for publication: *SHELXTL*.

## Supplementary Material

Crystal structure: contains datablocks global, I. DOI: 10.1107/S1600536808033758/lh2710sup1.cif

Structure factors: contains datablocks I. DOI: 10.1107/S1600536808033758/lh2710Isup2.hkl

Additional supplementary materials:  crystallographic information; 3D view; checkCIF report

## Figures and Tables

**Table 1:** Hydrogen-bond geometry (Å, °)

*D*—H⋯*A*	*D*—H	H⋯*A*	*D*⋯*A*	*D*—H⋯*A*
O3—H3*O*⋯O2	0.82 (3)	1.78 (2)	2.550 (4)	154 (4)

**Fig. 1.**
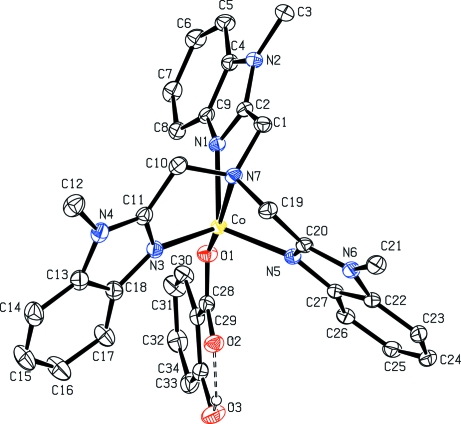
The cation of of the title compound with displacement ellipsoids shown at the 30% probability level. Hydrogen atoms bonded to C atoms are not shown and the dashed line indicates a hydrogen bond.

## supplementary crystallographic information

### Comment

The asymmetric unit of the title compound consists of a discrete [Co(Mentb)(salicylate)] cation (Fig. 1), a perchlorate anion and 1.5 molecules of DMF. The Co^II^ ion is five-coordinate with a N_4_O ligand set. The Mentb ligand acts as a tetradentate N-donor, and an O atom of carboxylate groups of the salicylate completes the coordination. The coordination geometry of the Co^II^ may be best described as distorted trigonal bipyramid (τ = 0.84), with approximat site symmetry C_3_. The parameter τ is defined as (β - α)/60 [where β = O1—Co—N7, α = N1—Co—N5] and its value varies from 0 (in regular square-based pyramidal) to 1 (in regular trigonal bipyramidal) [Youngme *et al.*, 2007]. This geometry is assumed by the Co^II^ to relieve the steric crowding. The equatorial plane is occupied by three N atoms of three benzimidazolyl groups, while the Co^II^ ion protrudes towards O1 and is 0.528 (2) Å from the plane of atoms N1/N3/N5. The axial positions are occupyied by N7 and O1. The three benzimidazole ring arms of the Mentb ligand form a cone-shaped cavity. The distance between Co^II^ and O2 is 3.167 (2) A, therefore O2 is not considered to be coordinated. The distances in the Mentb and salicylate are normally equal [Allen *et al.*, 1987].  There is a weak intramolecular O-H···O hydrogen bond and the crystal structure is stabilized by weak π···π stacking interactions with ring centroid···ring centroid distances of 3.623Å(1).

### Experimental

To a stirred solution of tris(*N*-methylbenzimidazol-2-ylmethyl)amine (0.0899 g, 0.2 mmol) in hot MeOH (10 ml) was added Co(ClO_4_)_2_ (H_2_O)_6_ (0.0732 g, 0.2 mmol), followed by a solution of Na(salicylate) (0.0320 g, 0.2 mmol) in MeOH (5 ml). A red crystalline product formed rapidly. The precipitate was filtered off, washed with MeOH and absolute Et_2_O, and dried *in vacuo*. The dried precipitate was dissolved in DMF to a red solution that was allowed to evaporate at room temperature. The red crystals suitable for X-ray diffraction studies were obtained after three weeks. Yield, 0.091 g (53%). (found: C, 54.23; H, 4.98; N,14.01. Calcd. for C38.50H42.50ClN8.50O8.50Co: C, 54.10; H, 5.01; N, 13.93)

### Refinement

All H atoms were found in difference electron maps and were subsequently refined in a riding-model approximation with C—H distances ranging from 0.95 to 0.99 Å with *U*_iso_(H) = 1.2 or 1.1 *U*_eq_(C) of the carrier atom. The H atom bonded to O3 was refined isotropically.

### Crystal data

**Table:**

[Co(C_7_H_5_O_3_)(C_27_H_27_N_7_)]ClO_4_·1.5C_3_H_7_NO	*F*(000) = 3560
*M**_r_* = 854.69	*D*_x_ = 1.467 Mg m^−^^3^
Monoclinic, *C*2/*c*	Mo *K*α radiation, λ = 0.71073 Å
Hall symbol: -C 2yc	Cell parameters from 8867 reflections
*a* = 27.7516 (5) Å	θ = 3.0–27.5°
*b* = 11.4051 (2) Å	µ = 0.58 mm^−^^1^
*c* = 25.0827 (5) Å	*T* = 153 K
β = 102.913 (1)°	Block, red
*V* = 7738.1 (2) Å^3^	0.35 × 0.25 × 0.21 mm
*Z* = 8	

### Data collection

**Table:**

Rigaku R-Axis Spider diffractometer	8867 independent reflections
Radiation source: rotating anode	6936 reflections with *I* > 2σ(*I*)
graphite	*R*_int_ = 0.029
ω scans	θ_max_ = 27.5°, θ_min_ = 3.0°
Absorption correction: empirical (using intensity measurements) (ABSCOR; Higashi, 1995)	*h* = −36→36
*T*_min_ = 0.823, *T*_max_ = 0.888	*k* = −13→14
36824 measured reflections	*l* = −32→32

### Refinement

**Table:**

Refinement on *F*^2^	Secondary atom site location: difference Fourier map
Least-squares matrix: full	Hydrogen site location: inferred from neighbouring sites
*R*[*F*^2^ > 2σ(*F*^2^)] = 0.039	H atoms treated by a mixture of independent and constrained refinement
*wR*(*F*^2^) = 0.121	*w* = 1/[σ^2^(*F*_o_^2^) + (0.068*P*)^2^ + 9.5996*P*] where *P* = (*F*_o_^2^ + 2*F*_c_^2^)/3
*S* = 1.00	(Δ/σ)_max_ = 0.003
8867 reflections	Δρ_max_ = 0.55 e Å^−^^3^
600 parameters	Δρ_min_ = −0.69 e Å^−^^3^
8 restraints	Extinction correction: SHELXL97 (Sheldrick, 2008), Fc^*^=kFc[1+0.001xFc^2^λ^3^/sin(2θ)]^-1/4^
Primary atom site location: structure-invariant direct methods	Extinction coefficient: 0.00040 (8)

### Special details

**Table:**

Geometry. All e.s.d.'s (except the e.s.d. in the dihedral angle between two l.s. planes) are estimated using the full covariance matrix. The cell e.s.d.'s are taken into account individually in the estimation of e.s.d.'s in distances, angles and torsion angles; correlations between e.s.d.'s in cell parameters are only used when they are defined by crystal symmetry. An approximate (isotropic) treatment of cell e.s.d.'s is used for estimating e.s.d.'s involving l.s. planes.
Refinement. Refinement of *F*^2^ against ALL reflections. The weighted *R*-factor *wR* and goodness of fit *S* are based on *F*^2^, conventional *R*-factors *R* are based on *F*, with *F* set to zero for negative *F*^2^. The threshold expression of *F*^2^ > σ(*F*^2^) is used only for calculating *R*-factors(gt) *etc*. and is not relevant to the choice of reflections for refinement. *R*-factors based on *F*^2^ are statistically about twice as large as those based on *F*, and *R*- factors based on ALL data will be even larger.

### Fractional atomic coordinates and isotropic or equivalent isotropic displacement parameters (Å^2^)

**Table:**

	*x*	*y*	*z*	*U*_iso_*/*U*_eq_	Occ. (<1)
Co	0.643165 (9)	0.84449 (2)	0.588680 (11)	0.02494 (9)	
Cl	0.55437 (2)	0.47251 (5)	0.39942 (3)	0.04547 (16)	
O1	0.68856 (6)	0.98332 (13)	0.60019 (7)	0.0343 (3)	
O2	0.63229 (6)	1.11734 (14)	0.60628 (7)	0.0363 (4)	
O3	0.65477 (7)	1.33445 (15)	0.61296 (9)	0.0483 (4)	
O4	0.5566 (2)	0.5994 (6)	0.4022 (3)	0.0536 (14)	0.50
O5	0.5532 (3)	0.4355 (6)	0.4534 (2)	0.0541 (15)	0.50
O4'	0.5477 (4)	0.5840 (9)	0.3758 (6)	0.170 (6)	0.50
O5'	0.5428 (4)	0.4645 (10)	0.4537 (2)	0.149 (6)	0.50
O6	0.51748 (9)	0.4069 (3)	0.36336 (10)	0.0897 (9)	
O7	0.60101 (8)	0.4325 (2)	0.39541 (13)	0.0805 (8)	
O8	0.69071 (10)	1.5259 (2)	0.73387 (11)	0.0745 (7)	
N1	0.69868 (6)	0.73142 (15)	0.57844 (7)	0.0268 (3)	
N2	0.72198 (7)	0.58434 (16)	0.53149 (7)	0.0308 (4)	
N3	0.61529 (6)	0.80894 (16)	0.65624 (7)	0.0298 (4)	
N4	0.58874 (7)	0.68285 (18)	0.71079 (8)	0.0354 (4)	
N5	0.58656 (6)	0.87214 (15)	0.52153 (7)	0.0270 (4)	
N6	0.51379 (6)	0.82005 (16)	0.46910 (8)	0.0310 (4)	
N7	0.60565 (6)	0.66102 (15)	0.57089 (7)	0.0282 (4)	
N8	0.71852 (8)	1.34132 (19)	0.75418 (9)	0.0435 (5)	
C1	0.63219 (8)	0.59755 (19)	0.53486 (9)	0.0309 (4)	
H1A	0.6169	0.6144	0.4961	0.037*	
H1B	0.6305	0.5120	0.5408	0.037*	
C2	0.68453 (8)	0.63739 (18)	0.54816 (9)	0.0280 (4)	
C3	0.71908 (9)	0.4812 (2)	0.49655 (10)	0.0392 (5)	
H3A	0.7280	0.4112	0.5193	0.047*	
H3B	0.7420	0.4901	0.4723	0.047*	
H3C	0.6853	0.4729	0.4746	0.047*	
C4	0.76444 (8)	0.64746 (18)	0.55368 (9)	0.0300 (4)	
C5	0.81369 (9)	0.6297 (2)	0.55124 (10)	0.0376 (5)	
H5	0.8234	0.5670	0.5310	0.045*	
C6	0.84761 (8)	0.7087 (2)	0.57998 (10)	0.0398 (5)	
H6	0.8816	0.6999	0.5795	0.048*	
C7	0.83338 (8)	0.8009 (2)	0.60954 (10)	0.0369 (5)	
H7	0.8578	0.8534	0.6286	0.044*	
C8	0.78428 (8)	0.81783 (19)	0.61179 (9)	0.0307 (4)	
H8	0.7748	0.8806	0.6321	0.037*	
C9	0.74962 (7)	0.73929 (18)	0.58308 (8)	0.0275 (4)	
C10	0.61014 (8)	0.6048 (2)	0.62462 (9)	0.0346 (5)	
H10A	0.6429	0.5668	0.6363	0.042*	
H10B	0.5842	0.5443	0.6227	0.042*	
C11	0.60428 (7)	0.69829 (19)	0.66407 (9)	0.0309 (4)	
C12	0.57561 (10)	0.5741 (2)	0.73431 (11)	0.0467 (6)	
H12A	0.5716	0.5117	0.7068	0.056*	
H12B	0.5445	0.5848	0.7461	0.056*	
H12C	0.6019	0.5525	0.7659	0.056*	
C13	0.59032 (8)	0.7915 (2)	0.73578 (10)	0.0368 (5)	
C14	0.57893 (10)	0.8257 (3)	0.78514 (11)	0.0483 (6)	
H14	0.5672	0.7713	0.8080	0.058*	
C15	0.58553 (12)	0.9414 (3)	0.79863 (12)	0.0591 (8)	
H15	0.5784	0.9683	0.8319	0.071*	
C16	0.60248 (12)	1.0213 (3)	0.76483 (12)	0.0583 (8)	
H16	0.6070	1.1009	0.7760	0.070*	
C17	0.61291 (10)	0.9877 (2)	0.71532 (11)	0.0444 (6)	
H17	0.6237	1.0429	0.6921	0.053*	
C18	0.60693 (8)	0.8704 (2)	0.70135 (9)	0.0344 (5)	
C19	0.55367 (7)	0.68142 (18)	0.54340 (9)	0.0311 (4)	
H19A	0.5332	0.6897	0.5708	0.037*	
H19B	0.5409	0.6145	0.5192	0.037*	
C20	0.55149 (7)	0.79069 (18)	0.51077 (8)	0.0281 (4)	
C21	0.47023 (8)	0.7489 (2)	0.44631 (10)	0.0385 (5)	
H21A	0.4767	0.7009	0.4163	0.046*	
H21B	0.4419	0.8004	0.4325	0.046*	
H21C	0.4629	0.6977	0.4749	0.046*	
C22	0.52446 (8)	0.92923 (19)	0.45085 (9)	0.0311 (4)	
C23	0.49798 (9)	1.0012 (2)	0.40966 (9)	0.0376 (5)	
H23	0.4666	0.9790	0.3882	0.045*	
C24	0.51983 (9)	1.1062 (2)	0.40184 (10)	0.0415 (6)	
H24	0.5030	1.1578	0.3741	0.050*	
C25	0.56612 (9)	1.1395 (2)	0.43353 (10)	0.0388 (5)	
H25	0.5800	1.2125	0.4265	0.047*	
C26	0.59188 (8)	1.06819 (19)	0.47479 (9)	0.0330 (5)	
H26	0.6232	1.0910	0.4963	0.040*	
C27	0.57031 (8)	0.96123 (18)	0.48382 (8)	0.0288 (4)	
C28	0.67583 (8)	1.08797 (19)	0.60662 (8)	0.0297 (4)	
C29	0.71489 (8)	1.18065 (18)	0.61520 (8)	0.0298 (4)	
C30	0.76472 (8)	1.1523 (2)	0.62112 (10)	0.0359 (5)	
H30	0.7739	1.0724	0.6195	0.043*	
C31	0.80089 (9)	1.2374 (2)	0.62929 (11)	0.0446 (6)	
H31	0.8345	1.2166	0.6323	0.053*	
C32	0.78756 (10)	1.3542 (2)	0.63310 (11)	0.0445 (6)	
H32	0.8123	1.4132	0.6392	0.053*	
C33	0.73888 (10)	1.3848 (2)	0.62805 (10)	0.0407 (5)	
H33	0.7302	1.4647	0.6311	0.049*	
C34	0.70224 (9)	1.29931 (19)	0.61845 (9)	0.0346 (5)	
C35	0.6705 (3)	1.2899 (6)	0.7445 (3)	0.0629 (17)	0.50
H35A	0.6455	1.3508	0.7329	0.075*	0.50
H35B	0.6656	1.2530	0.7781	0.075*	0.50
H35C	0.6675	1.2305	0.7157	0.075*	0.50
C36	0.7614 (3)	1.2675 (7)	0.7674 (3)	0.0648 (18)	0.50
H36A	0.7611	1.2126	0.7373	0.078*	0.50
H36B	0.7611	1.2236	0.8009	0.078*	0.50
H36C	0.7913	1.3160	0.7731	0.078*	0.50
C37	0.7221 (2)	1.4602 (5)	0.7472 (2)	0.0475 (12)	0.50
H37	0.7546	1.4915	0.7544	0.057*	0.50
C35'	0.7025 (3)	1.2156 (6)	0.7539 (3)	0.0683 (18)	0.50
H35D	0.6663	1.2114	0.7436	0.082*	0.50
H35E	0.7143	1.1819	0.7904	0.082*	0.50
H35F	0.7164	1.1714	0.7274	0.082*	0.50
C36'	0.7701 (2)	1.3623 (10)	0.7691 (3)	0.080 (3)	0.50
H36D	0.7766	1.4460	0.7651	0.097*	0.50
H36E	0.7867	1.3168	0.7454	0.097*	0.50
H36F	0.7828	1.3388	0.8073	0.097*	0.50
C37'	0.6846 (2)	1.4203 (5)	0.7383 (2)	0.0480 (12)	0.50
H37'	0.6514	1.3932	0.7292	0.058*	0.50
O9	0.5719 (3)	1.3091 (6)	0.7098 (5)	0.179 (5)	0.50
N9	0.5049 (3)	1.2538 (6)	0.7471 (4)	0.123 (4)	0.50
C38	0.5482 (3)	1.3038 (6)	0.7456 (5)	0.190 (10)	0.50
H38	0.5635	1.3427	0.7786	0.209*	0.50
C39	0.4840 (6)	1.1958 (13)	0.6957 (4)	0.160 (6)	0.50
H39A	0.4904	1.1114	0.6996	0.176*	0.50
H39B	0.4993	1.2272	0.6671	0.176*	0.50
H39C	0.4483	1.2098	0.6858	0.176*	0.50
C40	0.4795 (5)	1.2510 (9)	0.7916 (5)	0.178 (9)	0.50
H40A	0.4468	1.2154	0.7788	0.196*	0.50
H40B	0.4757	1.3311	0.8042	0.196*	0.50
H40C	0.4987	1.2046	0.8219	0.196*	0.50
H3O	0.6388 (14)	1.273 (2)	0.6093 (19)	0.105 (15)*	

### Atomic displacement parameters (Å^2^)

**Table:**

	*U*^11^	*U*^22^	*U*^33^	*U*^12^	*U*^13^	*U*^23^
Co	0.01968 (14)	0.02878 (15)	0.02593 (15)	0.00379 (10)	0.00417 (10)	−0.00125 (10)
Cl	0.0303 (3)	0.0401 (3)	0.0677 (4)	−0.0048 (2)	0.0147 (3)	−0.0054 (3)
O1	0.0290 (8)	0.0313 (8)	0.0419 (9)	0.0008 (6)	0.0066 (7)	−0.0045 (7)
O2	0.0282 (8)	0.0369 (8)	0.0412 (9)	0.0054 (6)	0.0021 (7)	−0.0030 (7)
O3	0.0406 (10)	0.0359 (9)	0.0617 (12)	0.0087 (8)	−0.0028 (9)	−0.0007 (8)
O4	0.039 (3)	0.039 (3)	0.088 (4)	0.009 (2)	0.026 (3)	−0.005 (2)
O5	0.073 (4)	0.059 (3)	0.034 (2)	−0.026 (3)	0.018 (2)	−0.005 (2)
O4'	0.071 (5)	0.055 (5)	0.360 (19)	−0.005 (3)	−0.008 (8)	0.070 (8)
O5'	0.096 (6)	0.219 (12)	0.165 (8)	−0.092 (7)	0.097 (6)	−0.155 (8)
O6	0.0636 (15)	0.137 (2)	0.0601 (15)	−0.0444 (16)	−0.0049 (12)	0.0005 (15)
O7	0.0498 (13)	0.0572 (13)	0.145 (3)	0.0048 (10)	0.0438 (15)	−0.0073 (14)
O8	0.0856 (18)	0.0598 (14)	0.0745 (16)	0.0111 (13)	0.0105 (14)	−0.0031 (12)
N1	0.0214 (8)	0.0309 (8)	0.0279 (9)	0.0041 (6)	0.0048 (7)	0.0005 (7)
N2	0.0303 (9)	0.0347 (9)	0.0282 (9)	0.0084 (7)	0.0081 (7)	−0.0008 (7)
N3	0.0260 (8)	0.0377 (9)	0.0261 (9)	0.0038 (7)	0.0067 (7)	0.0004 (7)
N4	0.0282 (9)	0.0460 (11)	0.0327 (10)	0.0032 (8)	0.0082 (8)	0.0073 (8)
N5	0.0216 (8)	0.0318 (9)	0.0277 (9)	0.0050 (6)	0.0056 (7)	−0.0001 (7)
N6	0.0215 (8)	0.0392 (10)	0.0309 (9)	0.0067 (7)	0.0027 (7)	−0.0035 (8)
N7	0.0219 (8)	0.0307 (9)	0.0312 (9)	0.0043 (6)	0.0043 (7)	−0.0005 (7)
N8	0.0383 (11)	0.0563 (13)	0.0327 (11)	0.0034 (9)	0.0014 (9)	−0.0020 (9)
C1	0.0275 (10)	0.0319 (10)	0.0326 (11)	0.0043 (8)	0.0048 (9)	−0.0049 (9)
C2	0.0261 (10)	0.0302 (10)	0.0270 (10)	0.0071 (8)	0.0043 (8)	0.0016 (8)
C3	0.0453 (13)	0.0380 (12)	0.0345 (12)	0.0099 (10)	0.0095 (10)	−0.0068 (10)
C4	0.0290 (10)	0.0343 (11)	0.0276 (10)	0.0059 (8)	0.0084 (8)	0.0039 (8)
C5	0.0335 (12)	0.0435 (12)	0.0401 (13)	0.0112 (9)	0.0175 (10)	0.0042 (10)
C6	0.0253 (10)	0.0526 (14)	0.0453 (14)	0.0070 (10)	0.0160 (10)	0.0086 (11)
C7	0.0266 (10)	0.0455 (12)	0.0389 (12)	−0.0012 (9)	0.0082 (9)	0.0080 (10)
C8	0.0276 (10)	0.0364 (11)	0.0288 (11)	0.0035 (8)	0.0081 (8)	0.0038 (9)
C9	0.0233 (9)	0.0329 (10)	0.0267 (10)	0.0066 (8)	0.0066 (8)	0.0065 (8)
C10	0.0329 (11)	0.0349 (11)	0.0359 (12)	0.0049 (9)	0.0076 (9)	0.0061 (9)
C11	0.0222 (9)	0.0385 (11)	0.0309 (11)	0.0040 (8)	0.0036 (8)	0.0046 (9)
C12	0.0444 (14)	0.0499 (14)	0.0482 (15)	0.0031 (11)	0.0155 (12)	0.0151 (12)
C13	0.0278 (11)	0.0500 (13)	0.0329 (12)	0.0034 (9)	0.0077 (9)	0.0017 (10)
C14	0.0449 (14)	0.0683 (17)	0.0354 (13)	0.0009 (12)	0.0168 (11)	0.0040 (12)
C15	0.074 (2)	0.0709 (19)	0.0400 (15)	−0.0026 (16)	0.0300 (15)	−0.0112 (14)
C16	0.076 (2)	0.0564 (17)	0.0509 (17)	−0.0079 (14)	0.0318 (16)	−0.0155 (13)
C17	0.0511 (15)	0.0488 (14)	0.0379 (13)	−0.0048 (11)	0.0197 (12)	−0.0071 (11)
C18	0.0273 (10)	0.0470 (13)	0.0298 (11)	0.0035 (9)	0.0079 (9)	−0.0007 (9)
C19	0.0216 (9)	0.0326 (10)	0.0374 (12)	0.0009 (8)	0.0029 (8)	−0.0009 (9)
C20	0.0203 (9)	0.0343 (10)	0.0293 (10)	0.0060 (8)	0.0049 (8)	−0.0036 (8)
C21	0.0221 (10)	0.0480 (13)	0.0413 (13)	0.0037 (9)	−0.0018 (9)	−0.0089 (10)
C22	0.0271 (10)	0.0389 (11)	0.0277 (10)	0.0117 (8)	0.0070 (8)	−0.0015 (9)
C23	0.0321 (11)	0.0519 (14)	0.0281 (11)	0.0171 (10)	0.0049 (9)	0.0002 (10)
C24	0.0459 (14)	0.0485 (13)	0.0315 (12)	0.0238 (11)	0.0116 (10)	0.0068 (10)
C25	0.0492 (14)	0.0357 (12)	0.0357 (12)	0.0135 (10)	0.0185 (11)	0.0035 (9)
C26	0.0332 (11)	0.0352 (11)	0.0328 (11)	0.0063 (9)	0.0120 (9)	−0.0020 (9)
C27	0.0270 (10)	0.0348 (10)	0.0254 (10)	0.0105 (8)	0.0079 (8)	−0.0012 (8)
C28	0.0276 (10)	0.0351 (11)	0.0250 (10)	0.0029 (8)	0.0030 (8)	−0.0014 (8)
C29	0.0327 (11)	0.0336 (10)	0.0223 (10)	0.0017 (8)	0.0042 (8)	−0.0011 (8)
C30	0.0337 (11)	0.0384 (12)	0.0370 (12)	−0.0007 (9)	0.0108 (10)	−0.0087 (9)
C31	0.0360 (12)	0.0503 (14)	0.0491 (15)	−0.0065 (10)	0.0131 (11)	−0.0109 (12)
C32	0.0498 (15)	0.0435 (13)	0.0403 (14)	−0.0128 (11)	0.0103 (11)	−0.0028 (11)
C33	0.0522 (15)	0.0318 (11)	0.0356 (12)	−0.0020 (10)	0.0042 (11)	0.0027 (10)
C34	0.0415 (12)	0.0332 (11)	0.0258 (11)	0.0040 (9)	0.0008 (9)	0.0012 (9)
C35	0.073 (4)	0.062 (4)	0.052 (4)	−0.028 (3)	0.011 (3)	−0.011 (3)
C36	0.074 (5)	0.072 (4)	0.048 (4)	0.027 (4)	0.013 (3)	0.007 (3)
C37	0.047 (3)	0.044 (3)	0.047 (3)	−0.005 (2)	0.000 (2)	−0.001 (2)
C35'	0.106 (6)	0.055 (4)	0.044 (3)	0.011 (4)	0.019 (4)	−0.004 (3)
C36'	0.027 (3)	0.170 (9)	0.040 (3)	0.015 (4)	−0.001 (2)	−0.005 (5)
C37'	0.035 (3)	0.059 (3)	0.046 (3)	0.002 (2)	0.002 (2)	−0.006 (3)
O9	0.075 (5)	0.085 (4)	0.389 (16)	−0.020 (4)	0.079 (7)	0.035 (7)
N9	0.161 (9)	0.078 (4)	0.111 (7)	0.050 (8)	−0.012 (7)	0.027 (8)
C38	0.045 (5)	0.052 (5)	0.47 (3)	−0.007 (4)	0.040 (10)	0.051 (9)
C39	0.187 (14)	0.228 (15)	0.076 (7)	−0.104 (12)	0.051 (8)	−0.026 (8)
C40	0.129 (13)	0.099 (9)	0.24 (2)	0.009 (8)	−0.107 (14)	−0.014 (10)

### Geometric parameters (Å, °)

**Table:**

Co—O1	2.0038 (15)	C13—C18	1.395 (3)
Co—N3	2.0543 (17)	C13—C14	1.400 (3)
Co—N5	2.0546 (17)	C14—C15	1.364 (4)
Co—N1	2.0687 (16)	C14—H14	0.9500
Co—N7	2.3353 (17)	C15—C16	1.396 (4)
Cl—O7	1.397 (2)	C15—H15	0.9500
Cl—O4'	1.398 (10)	C16—C17	1.390 (3)
Cl—O6	1.419 (2)	C16—H16	0.9500
Cl—O5	1.424 (4)	C17—C18	1.384 (3)
Cl—O4	1.450 (7)	C17—H17	0.9500
Cl—O5'	1.470 (3)	C19—C20	1.485 (3)
O1—C28	1.265 (3)	C19—H19A	0.9900
O2—C28	1.252 (3)	C19—H19B	0.9900
O3—C34	1.354 (3)	C21—H21A	0.9800
O3—H3O	0.82 (3)	C21—H21B	0.9800
O8—C37	1.142 (6)	C21—H21C	0.9800
O8—C37'	1.225 (6)	C22—C23	1.394 (3)
N1—C2	1.322 (3)	C22—C27	1.402 (3)
N1—C9	1.395 (2)	C23—C24	1.376 (4)
N2—C2	1.347 (3)	C23—H23	0.9500
N2—C4	1.387 (3)	C24—C25	1.404 (4)
N2—C3	1.458 (3)	C24—H24	0.9500
N3—C11	1.323 (3)	C25—C26	1.383 (3)
N3—C18	1.394 (3)	C25—H25	0.9500
N4—C11	1.348 (3)	C26—C27	1.399 (3)
N4—C13	1.385 (3)	C26—H26	0.9500
N4—C12	1.454 (3)	C28—C29	1.495 (3)
N5—C20	1.329 (3)	C29—C30	1.396 (3)
N5—C27	1.393 (3)	C29—C34	1.405 (3)
N6—C20	1.346 (3)	C30—C31	1.378 (3)
N6—C22	1.381 (3)	C30—H30	0.9500
N6—C21	1.462 (3)	C31—C32	1.391 (4)
N7—C19	1.472 (3)	C31—H31	0.9500
N7—C10	1.472 (3)	C32—C33	1.373 (4)
N7—C1	1.477 (3)	C32—H32	0.9500
N8—C37'	1.300 (6)	C33—C34	1.390 (3)
N8—C37	1.373 (6)	C33—H33	0.9500
N8—C36'	1.418 (7)	C35—H35A	0.9800
N8—C35	1.426 (6)	C35—H35B	0.9800
N8—C36	1.435 (7)	C35—H35C	0.9800
N8—C35'	1.501 (7)	C36—H36A	0.9800
C1—C2	1.487 (3)	C36—H36B	0.9800
C1—H1A	0.9900	C36—H36C	0.9800
C1—H1B	0.9900	C37—H37	0.9500
C3—H3A	0.9800	C35'—H35D	0.9800
C3—H3B	0.9800	C35'—H35E	0.9800
C3—H3C	0.9800	C35'—H35F	0.9800
C4—C9	1.395 (3)	C36'—H36D	0.9800
C4—C5	1.397 (3)	C36'—H36E	0.9800
C5—C6	1.383 (4)	C36'—H36F	0.9800
C5—H5	0.9500	C37'—H37'	0.9500
C6—C7	1.393 (3)	O9—C38	1.229 (3)
C6—H6	0.9500	N9—C38	1.338 (3)
C7—C8	1.390 (3)	N9—C40	1.448 (3)
C7—H7	0.9500	N9—C39	1.450 (3)
C8—C9	1.392 (3)	C38—H38	0.9500
C8—H8	0.9500	C39—H39A	0.9800
C10—C11	1.488 (3)	C39—H39B	0.9800
C10—H10A	0.9900	C39—H39C	0.9800
C10—H10B	0.9900	C40—H40A	0.9800
C12—H12A	0.9800	C40—H40B	0.9800
C12—H12B	0.9800	C40—H40C	0.9800
C12—H12C	0.9800		
			
O1—Co—N3	112.19 (7)	N4—C13—C14	131.2 (2)
O1—Co—N5	109.74 (7)	C18—C13—C14	122.6 (2)
N3—Co—N5	110.28 (7)	C15—C14—C13	116.4 (2)
O1—Co—N1	92.79 (6)	C15—C14—H14	121.8
N3—Co—N1	114.05 (7)	C13—C14—H14	121.8
N5—Co—N1	116.62 (7)	C14—C15—C16	121.7 (2)
O1—Co—N7	167.07 (6)	C14—C15—H15	119.1
N3—Co—N7	75.33 (7)	C16—C15—H15	119.1
N5—Co—N7	75.78 (7)	C17—C16—C15	121.9 (3)
N1—Co—N7	74.35 (6)	C17—C16—H16	119.1
O7—Cl—O4'	107.6 (5)	C15—C16—H16	119.1
O7—Cl—O6	109.26 (18)	C18—C17—C16	117.1 (2)
O4'—Cl—O6	101.7 (5)	C18—C17—H17	121.5
O7—Cl—O5	100.9 (4)	C16—C17—H17	121.5
O4'—Cl—O5	130.0 (7)	C17—C18—N3	131.3 (2)
O6—Cl—O5	106.4 (3)	C17—C18—C13	120.3 (2)
O7—Cl—O4	107.4 (2)	N3—C18—C13	108.4 (2)
O6—Cl—O4	125.1 (3)	N7—C19—C20	107.80 (16)
O5—Cl—O4	105.1 (4)	N7—C19—H19A	110.1
O7—Cl—O5'	116.4 (5)	C20—C19—H19A	110.1
O4'—Cl—O5'	114.4 (8)	N7—C19—H19B	110.1
O6—Cl—O5'	106.4 (4)	C20—C19—H19B	110.1
O4—Cl—O5'	91.9 (5)	H19A—C19—H19B	108.5
C28—O1—Co	125.40 (14)	N5—C20—N6	113.01 (19)
C34—O3—H3O	104 (3)	N5—C20—C19	122.49 (19)
C37—O8—C37'	55.9 (4)	N6—C20—C19	124.46 (19)
C2—N1—C9	105.39 (16)	N6—C21—H21A	109.5
C2—N1—Co	116.30 (13)	N6—C21—H21B	109.5
C9—N1—Co	135.93 (14)	H21A—C21—H21B	109.5
C2—N2—C4	106.70 (17)	N6—C21—H21C	109.5
C2—N2—C3	127.30 (19)	H21A—C21—H21C	109.5
C4—N2—C3	126.00 (18)	H21B—C21—H21C	109.5
C11—N3—C18	105.86 (18)	N6—C22—C23	131.3 (2)
C11—N3—Co	116.92 (14)	N6—C22—C27	105.83 (18)
C18—N3—Co	137.05 (15)	C23—C22—C27	122.9 (2)
C11—N4—C13	107.02 (19)	C24—C23—C22	116.2 (2)
C11—N4—C12	128.5 (2)	C24—C23—H23	121.9
C13—N4—C12	124.4 (2)	C22—C23—H23	121.9
C20—N5—C27	105.11 (17)	C23—C24—C25	122.1 (2)
C20—N5—Co	116.76 (14)	C23—C24—H24	118.9
C27—N5—Co	137.81 (15)	C25—C24—H24	118.9
C20—N6—C22	107.13 (18)	C26—C25—C24	121.3 (2)
C20—N6—C21	126.7 (2)	C26—C25—H25	119.4
C22—N6—C21	126.20 (19)	C24—C25—H25	119.4
C19—N7—C10	111.79 (17)	C25—C26—C27	117.7 (2)
C19—N7—C1	111.19 (17)	C25—C26—H26	121.1
C10—N7—C1	113.26 (16)	C27—C26—H26	121.1
C19—N7—Co	107.24 (12)	N5—C27—C26	131.3 (2)
C10—N7—Co	105.81 (13)	N5—C27—C22	108.91 (19)
C1—N7—Co	107.11 (12)	C26—C27—C22	119.7 (2)
C37'—N8—C37	49.1 (4)	O2—C28—O1	123.2 (2)
C37'—N8—C36'	125.7 (5)	O2—C28—C29	118.65 (19)
C37—N8—C36'	76.7 (5)	O1—C28—C29	118.11 (18)
C37'—N8—C35	69.4 (4)	C30—C29—C34	118.1 (2)
C37—N8—C35	118.4 (4)	C30—C29—C28	121.39 (19)
C36'—N8—C35	164.8 (6)	C34—C29—C28	120.5 (2)
C37'—N8—C36	169.8 (5)	C31—C30—C29	121.6 (2)
C37—N8—C36	121.9 (5)	C31—C30—H30	119.2
C36'—N8—C36	45.7 (4)	C29—C30—H30	119.2
C35—N8—C36	119.6 (5)	C30—C31—C32	119.2 (2)
C37'—N8—C35'	117.8 (4)	C30—C31—H31	120.4
C37—N8—C35'	166.5 (4)	C32—C31—H31	120.4
C36'—N8—C35'	116.3 (6)	C33—C32—C31	120.5 (2)
C35—N8—C35'	48.8 (4)	C33—C32—H32	119.7
C36—N8—C35'	70.8 (5)	C31—C32—H32	119.7
N7—C1—C2	107.99 (17)	C32—C33—C34	120.3 (2)
N7—C1—H1A	110.1	C32—C33—H33	119.9
C2—C1—H1A	110.1	C34—C33—H33	119.9
N7—C1—H1B	110.1	O3—C34—C33	117.9 (2)
C2—C1—H1B	110.1	O3—C34—C29	121.9 (2)
H1A—C1—H1B	108.4	C33—C34—C29	120.2 (2)
N1—C2—N2	113.07 (18)	N8—C35—H35A	109.5
N1—C2—C1	121.77 (17)	N8—C35—H35B	109.5
N2—C2—C1	125.16 (19)	N8—C35—H35C	109.5
N2—C3—H3A	109.5	N8—C36—H36A	109.5
N2—C3—H3B	109.5	N8—C36—H36B	109.5
H3A—C3—H3B	109.5	N8—C36—H36C	109.5
N2—C3—H3C	109.5	O8—C37—N8	127.9 (5)
H3A—C3—H3C	109.5	O8—C37—H37	116.1
H3B—C3—H3C	109.5	N8—C37—H37	116.1
N2—C4—C9	106.12 (17)	N8—C35'—H35D	109.5
N2—C4—C5	131.3 (2)	N8—C35'—H35E	109.5
C9—C4—C5	122.6 (2)	H35D—C35'—H35E	109.5
C6—C5—C4	116.2 (2)	N8—C35'—H35F	109.5
C6—C5—H5	121.9	H35D—C35'—H35F	109.5
C4—C5—H5	121.9	H35E—C35'—H35F	109.5
C5—C6—C7	121.9 (2)	N8—C36'—H36D	109.5
C5—C6—H6	119.1	N8—C36'—H36E	109.5
C7—C6—H6	119.1	H36D—C36'—H36E	109.5
C8—C7—C6	121.6 (2)	N8—C36'—H36F	109.5
C8—C7—H7	119.2	H36D—C36'—H36F	109.5
C6—C7—H7	119.2	H36E—C36'—H36F	109.5
C7—C8—C9	117.4 (2)	O8—C37'—N8	127.1 (5)
C7—C8—H8	121.3	O8—C37'—H37'	116.4
C9—C8—H8	121.3	N8—C37'—H37'	116.4
C8—C9—C4	120.37 (18)	C38—N9—C40	128.8 (11)
C8—C9—N1	130.91 (19)	C38—N9—C39	110.7 (11)
C4—C9—N1	108.70 (18)	C40—N9—C39	120.5 (9)
N7—C10—C11	107.28 (17)	O9—C38—N9	132.3 (10)
N7—C10—H10A	110.3	O9—C38—H38	113.9
C11—C10—H10A	110.3	N9—C38—H38	113.9
N7—C10—H10B	110.3	N9—C39—H39A	109.5
C11—C10—H10B	110.3	N9—C39—H39B	109.5
H10A—C10—H10B	108.5	H39A—C39—H39B	109.5
N3—C11—N4	112.5 (2)	N9—C39—H39C	109.5
N3—C11—C10	121.43 (19)	H39A—C39—H39C	109.5
N4—C11—C10	126.0 (2)	H39B—C39—H39C	109.5
N4—C12—H12A	109.5	N9—C40—H40A	109.5
N4—C12—H12B	109.5	N9—C40—H40B	109.5
H12A—C12—H12B	109.5	H40A—C40—H40B	109.5
N4—C12—H12C	109.5	N9—C40—H40C	109.5
H12A—C12—H12C	109.5	H40A—C40—H40C	109.5
H12B—C12—H12C	109.5	H40B—C40—H40C	109.5
N4—C13—C18	106.18 (19)		
			
N3—Co—O1—C28	−66.15 (18)	N7—C10—C11—N4	155.0 (2)
N5—Co—O1—C28	56.81 (18)	C11—N4—C13—C18	0.6 (2)
N1—Co—O1—C28	176.42 (17)	C12—N4—C13—C18	176.9 (2)
N7—Co—O1—C28	170.2 (3)	C11—N4—C13—C14	−179.0 (3)
O1—Co—N1—C2	−153.11 (15)	C12—N4—C13—C14	−2.7 (4)
N3—Co—N1—C2	91.05 (16)	N4—C13—C14—C15	178.7 (3)
N5—Co—N1—C2	−39.37 (17)	C18—C13—C14—C15	−0.9 (4)
N7—Co—N1—C2	25.44 (15)	C13—C14—C15—C16	0.3 (5)
O1—Co—N1—C9	6.3 (2)	C14—C15—C16—C17	0.9 (5)
N3—Co—N1—C9	−109.53 (19)	C15—C16—C17—C18	−1.5 (5)
N5—Co—N1—C9	120.05 (19)	C16—C17—C18—N3	−178.1 (3)
N7—Co—N1—C9	−175.1 (2)	C16—C17—C18—C13	1.0 (4)
O1—Co—N3—C11	−150.67 (15)	C11—N3—C18—C17	178.9 (3)
N5—Co—N3—C11	86.68 (16)	Co—N3—C18—C17	4.1 (4)
N1—Co—N3—C11	−46.80 (17)	C11—N3—C18—C13	−0.3 (2)
N7—Co—N3—C11	18.23 (15)	Co—N3—C18—C13	−175.14 (16)
O1—Co—N3—C18	23.7 (2)	N4—C13—C18—C17	−179.5 (2)
N5—Co—N3—C18	−98.9 (2)	C14—C13—C18—C17	0.2 (4)
N1—Co—N3—C18	127.6 (2)	N4—C13—C18—N3	−0.1 (2)
N7—Co—N3—C18	−167.4 (2)	C14—C13—C18—N3	179.5 (2)
O1—Co—N5—C20	−175.68 (13)	C10—N7—C19—C20	146.98 (18)
N3—Co—N5—C20	−51.60 (16)	C1—N7—C19—C20	−85.3 (2)
N1—Co—N5—C20	80.57 (15)	Co—N7—C19—C20	31.44 (19)
N7—Co—N5—C20	16.56 (14)	C27—N5—C20—N6	−0.1 (2)
O1—Co—N5—C27	−3.2 (2)	Co—N5—C20—N6	174.69 (13)
N3—Co—N5—C27	120.84 (19)	C27—N5—C20—C19	−177.83 (18)
N1—Co—N5—C27	−107.00 (19)	Co—N5—C20—C19	−3.1 (2)
N7—Co—N5—C27	−171.0 (2)	C22—N6—C20—N5	−0.2 (2)
O1—Co—N7—C19	−143.9 (3)	C21—N6—C20—N5	178.40 (19)
N3—Co—N7—C19	88.90 (14)	C22—N6—C20—C19	177.54 (19)
N5—Co—N7—C19	−26.95 (13)	C21—N6—C20—C19	−3.9 (3)
N1—Co—N7—C19	−150.39 (14)	N7—C19—C20—N5	−21.8 (3)
O1—Co—N7—C10	96.7 (3)	N7—C19—C20—N6	160.68 (18)
N3—Co—N7—C10	−30.56 (12)	C20—N6—C22—C23	−178.7 (2)
N5—Co—N7—C10	−146.40 (13)	C21—N6—C22—C23	2.7 (4)
N1—Co—N7—C10	90.16 (13)	C20—N6—C22—C27	0.3 (2)
O1—Co—N7—C1	−24.4 (4)	C21—N6—C22—C27	−178.25 (19)
N3—Co—N7—C1	−151.66 (14)	N6—C22—C23—C24	−179.7 (2)
N5—Co—N7—C1	92.50 (14)	C27—C22—C23—C24	1.4 (3)
N1—Co—N7—C1	−30.94 (13)	C22—C23—C24—C25	−0.1 (3)
C19—N7—C1—C2	147.90 (17)	C23—C24—C25—C26	−0.7 (3)
C10—N7—C1—C2	−85.2 (2)	C24—C25—C26—C27	0.2 (3)
Co—N7—C1—C2	31.04 (19)	C20—N5—C27—C26	−179.0 (2)
C9—N1—C2—N2	−1.4 (2)	Co—N5—C27—C26	8.0 (3)
Co—N1—C2—N2	163.89 (13)	C20—N5—C27—C22	0.3 (2)
C9—N1—C2—C1	178.62 (19)	Co—N5—C27—C22	−172.74 (15)
Co—N1—C2—C1	−16.1 (3)	C25—C26—C27—N5	−179.8 (2)
C4—N2—C2—N1	1.4 (2)	C25—C26—C27—C22	1.0 (3)
C3—N2—C2—N1	−178.2 (2)	N6—C22—C27—N5	−0.4 (2)
C4—N2—C2—C1	−178.7 (2)	C23—C22—C27—N5	178.79 (18)
C3—N2—C2—C1	1.8 (3)	N6—C22—C27—C26	178.98 (17)
N7—C1—C2—N1	−13.2 (3)	C23—C22—C27—C26	−1.9 (3)
N7—C1—C2—N2	166.79 (19)	Co—O1—C28—O2	−0.3 (3)
C2—N2—C4—C9	−0.7 (2)	Co—O1—C28—C29	179.49 (14)
C3—N2—C4—C9	178.8 (2)	O2—C28—C29—C30	173.9 (2)
C2—N2—C4—C5	177.7 (2)	O1—C28—C29—C30	−5.9 (3)
C3—N2—C4—C5	−2.8 (4)	O2—C28—C29—C34	−5.0 (3)
N2—C4—C5—C6	−178.0 (2)	O1—C28—C29—C34	175.2 (2)
C9—C4—C5—C6	0.2 (3)	C34—C29—C30—C31	−0.8 (3)
C4—C5—C6—C7	−0.2 (4)	C28—C29—C30—C31	−179.7 (2)
C5—C6—C7—C8	0.2 (4)	C29—C30—C31—C32	1.6 (4)
C6—C7—C8—C9	−0.3 (3)	C30—C31—C32—C33	−0.9 (4)
C7—C8—C9—C4	0.3 (3)	C31—C32—C33—C34	−0.7 (4)
C7—C8—C9—N1	178.3 (2)	C32—C33—C34—O3	−179.0 (2)
N2—C4—C9—C8	178.29 (19)	C32—C33—C34—C29	1.6 (4)
C5—C4—C9—C8	−0.3 (3)	C30—C29—C34—O3	179.8 (2)
N2—C4—C9—N1	−0.1 (2)	C28—C29—C34—O3	−1.3 (3)
C5—C4—C9—N1	−178.68 (19)	C30—C29—C34—C33	−0.9 (3)
C2—N1—C9—C8	−177.3 (2)	C28—C29—C34—C33	178.1 (2)
Co—N1—C9—C8	21.8 (3)	C37'—O8—C37—N8	−1.2 (5)
C2—N1—C9—C4	0.9 (2)	C37'—N8—C37—O8	1.3 (5)
Co—N1—C9—C4	−160.00 (16)	C36'—N8—C37—O8	−178.3 (7)
C19—N7—C10—C11	−80.0 (2)	C35—N8—C37—O8	−0.6 (8)
C1—N7—C10—C11	153.41 (17)	C36—N8—C37—O8	175.4 (6)
Co—N7—C10—C11	36.38 (18)	C35'—N8—C37—O8	17 (2)
C18—N3—C11—N4	0.7 (2)	C37—O8—C37'—N8	1.3 (5)
Co—N3—C11—N4	176.74 (14)	C37—N8—C37'—O8	−1.2 (5)
C18—N3—C11—C10	−178.19 (19)	C36'—N8—C37'—O8	−0.7 (8)
Co—N3—C11—C10	−2.1 (3)	C35—N8—C37'—O8	177.1 (7)
C13—N4—C11—N3	−0.8 (2)	C36—N8—C37'—O8	−31 (3)
C12—N4—C11—N3	−177.0 (2)	C35'—N8—C37'—O8	−177.0 (5)
C13—N4—C11—C10	178.0 (2)	C40—N9—C38—O9	179.5 (4)
C12—N4—C11—C10	1.9 (4)	C39—N9—C38—O9	0.4 (4)
N7—C10—C11—N3	−26.2 (3)		

### Hydrogen-bond geometry (Å, °)

**Table:**

*D*—H···*A*	*D*—H	H···*A*	*D*···*A*	*D*—H···*A*
O3—H3O···O2	0.82 (3)	1.78 (2)	2.550 (4)	154 (4)
